# Anti-gp41 antibodies inhibit infection and transcytosis of HIV-1 infectious molecular clones expressing transmitted/founder envelopes

**DOI:** 10.1186/1742-4690-9-S2-P195

**Published:** 2012-09-13

**Authors:** S Jain, C Ochsenbauer, JC Kappes, KL Rosenthal

**Affiliations:** 1 University of Alabama at Birmingham, Birmingham, AL, USA; 2 McMaster University, Hamilton, Canada

## Background

Prophylactic vaccine strategies against HIV-1 must effectively prevent virus transmission, infection and cell-to-cell spread during the earliest stages of acute infection. Since the genital mucosa is the primary site of entry, mucosal defense is critical for early control of infection. Recent identification of transmitted/founder (T/F) HIV-1 genomes has demonstrated a consistent genetic bottleneck during mucosal transmission and suggests that T/F viruses may exhibit distinct phenotypes. HIV-1 Env gp41-specific responses are among the first to be generated in natural HIV infection.

## Methods

Previously, we developed chimeric virus-like particle (VLP) immunogens that elicit potent systemic and mucosal antibodies against two highly conserved regions of gp41, by employing an optimized immunization strategy. The ELDKWA and QARVLAVERY epitopes are found with the membrane proximal external region (MPER) and the coiled coil region of gp41, respectively. Importantly, the epitope-specific IgG and IgA fractions derived from immunized mice were shown to be effective in neutralizing and preventing transcytosis of HIV in vitro. In particular, the QARVLAVERY epitope is remarkably conserved and induced unusually high and early levels of anti-QARV IgA, making it an attractive candidate for generation of broadly reactive mucosal antibodies.

## Results

In this study, we assessed the effectiveness of mucosal and systemic mouse antibodies elicited against these gp41 epitopes to inhibit T/F Env function. For this, we employed recombinant infectious molecular clones (Env-IMC) of HIV-1 that encode mucosally transmitted/founder env genes. Our results show that the gp41-specific IgG and IgA fractions effectively prevented the infection of TZM-bl cells and inhibited HIV transcytosis in an assay measuring the passage of infectious virus across an epithelial monolayer. Interestingly, the T/F Env-IMC tested were more sensitive to the antibodies than the R5 lab-adapted strains included as controls.

## Conclusion

These results highlight the potential of gp41-based immunogens to impart effective mucosal protection in the earliest stages of HIV transmission and infection.

